# Prognostic values of tumoral MMP2 and MMP9 overexpression in breast cancer: a systematic review and meta-analysis

**DOI:** 10.1186/s12885-021-07860-2

**Published:** 2021-02-10

**Authors:** Hanfang Jiang, Huiping Li

**Affiliations:** Key Laboratory of Carcinogenesis and Translational Research (Ministry of Education/Beijing), Department of Breast Oncology, Peking University Cancer Hospital & Institute, No. 52nd Fucheng Road, Haidian District, Beijing, 100142 China

**Keywords:** Breast cancer, MMP2, MMP9, Survival, Meta-analysis

## Abstract

**Background:**

Breast cancer (BC) is a leading cause of cancer-related death in females worldwide. Previous studies have demonstrated that matrix metalloproteinases (MMPs) play key roles in metastasis and are associated with survival in various cancers. The prognostic values of MMP2 and MMP9 expression in BC have been investigated, but the results remain controversial. Thus, we performed the present meta-analysis to investigate the associations between MMP2/9 expressions in tumor cells with clinicopathologic features and survival outcome in BC patients.

**Methods:**

Eligible studies were searched in PubMed, Web of Science, EMBASE, CNKI and Wanfang databases. The associations of MMP2/9 overexpression in tumor cells with overall survival (OS), disease-free survival (DFS) and recurrence-free survival (RFS) were assessed by hazard ratio (HR) and 95% confidence interval (CI). The associations of MMP2/9 overexpression with clinicopathological features were investigated by calculating odds ratio (OR) and 95% CI. Subgroup analysis, sensitivity analysis, meta-regression, and analysis for publication bias were performed.

**Results:**

A total of 41 studies comprising 6517 patients with primary BC were finally included. MMP2 overexpression was associated with an unfavorable OS (HR = 1.60, 95% CI 1.33 –1.94, *P* < 0.001) while MMP9 overexpression predicted a shorter OS (HR = 1.52, 95% CI 1.30 –1.77, *P* < 0.001). MMP2 overexpression conferred a higher risk to distant metastasis (OR = 2.69, 95% CI 1.35–5.39, *P* = 0.005) and MMP9 overexpression correlated with lymph node metastasis (OR = 2.90, 95% CI 1.86 – 4.53, *P* < 0.001). Moreover, MMP2 and MMP9 overexpression were both associated with higher clinical stage and histological grade in BC patients. MMP9 overexpression was more frequent in patients with larger tumor sizes.

**Conclusions:**

Tumoral MMP2 and MMP9 are promising markers for predicting the prognosis in patients with BC.

**Supplementary Information:**

The online version contains supplementary material available at 10.1186/s12885-021-07860-2.

## Background

Breast cancer (BC) is the most prevalent malignancy and one of the leading causes of cancer-related death among females worldwide [1]. It accounts for 24.2% of newly diagnosed cancer cases and 15.0% of death from cancer in women [1]. Furthermore, the incidence and mortality rates of BC have been increasing in recent years [2]. Previous studies have identified metastasis, tumor stage, histological grade, expression of estrogen receptor (ER), progesterone receptor (PR), and human epidermal growth factor receptor 2 (HER2) as prognostic factors for BC [3]. The significant findings of these biomarkers have promoted the development of molecular-targeted therapy of BC [4]. Therefore, identifying more and more molecular biomarkers would definitely improve the treatment and management of BC in the future.

Matrix metalloproteinases (MMPs) are a group of zinc endopeptidases critical for the decomposition of extracellular matrix (ECM) components and basement membrane (BM) [5]. MMPs thus play pivotal roles in various physiological and pathological processes, including morphogenesis, wound healing, inflammation, cancer invasion, and metastasis [6]. MMPs are structurally divided into several subtypes, among which MMP2 and MMP9 belong to the gelatinase family that mainly degrades gelatin, collagens IV and V in ECM and BM through their proteolytic function [7]. In cancer, the overproduction or increased activity of MMP2/9 leads to the degradation of ECM and BM, allowing for the invasion of tumor cells to other tissues and tumor cell metastasis to distant organs [8]. MMP2/9 have also been implicated in cancer development and progression through their functions in cell apoptosis, proliferation, and angiogenesis [9–11].

Previous studies demonstrated that MMP2/9 are important prognostic factors for various cancers. MMP2 and MMP9 overexpression was associated with poor prognosis in oral cancers [12], retinoblastoma [13], bladder cancer [14], and ovarian epithelial cancer [15]. The prognostic value of MMP2/9 in BC has also been investigated. Several studies reported that MMP2/9 overexpression was related to clinicopathological characteristics and associated with poor survival in patients with BC [16–19], indicating that MMP2/9 may function as good prognostic markers for BC. However, other studies showed no associations of MMP2/9 overexpression with survival [20–23]. Thus, the associations between MMP2/9 expression and clinicopathological features and survival in BC remain controversial.

To evaluate the prognostic values of MMP2/9 in BC, we performed a meta-analysis for the associations between MMP2/9 overexpression in tumor cells with the clinicopathologic features and survival outcomes in BC patients.

## Methods

### Literature search strategy

This was a Preferred Reporting Items for Systematic Reviews and Meta-Analysis (PRISMA) statement based meta-analysis [24]. A comprehensive literature search was performed in PubMed, Web of Science, EMBASE, CNKI and Wanfang databases from study inception to June 30, 2020. The following search terms were used: (breast cancer OR breast tumor OR breast neoplasm OR breast carcinoma) AND (matrix metalloproteinase OR MMP2 OR MMP9 OR gelatinase). There was no language restriction. References in relevant articles were furtherly scanned for more potentially eligible studies.

### Inclusion and exclusion criteria

Studies that met the following criteria were included: 1) detecting the protein expression of MMP2 and/or MMP9 in tumor cells of breast cancer tissue by immunohistochemistry (IHC); 2) investigating the associations between MMP2/MMP9 overexpression and survival and/or clinicopathological features; 3) reporting hazard ratio (HR) with corresponding 95% confidence interval (CI) or survival curves for survival analysis, or providing sufficient data to calculate the odds ratio (OR) with 95% CI for clinicopathological features. Studies that measured mRNA expression or protein levels in serum or stromal cells were excluded. Reviews, meta-analyses and studies lacking sufficient data were excluded. If several studies had overlapping samples, only the largest one was included.

### Data extraction and quality assessment

The following data were extracted by two independent researchers: first author, publication year, country, sample size, follow-up duration, percent of infiltrating ductal carcinoma (IDC), criteria for MMPs overexpression, survival outcomes, HR and 95% CI, clinicopathological features, tissue and antibody used for IHC staining, and the data for the calculation of OR and 95% CI. The quality of included studies were assessed by Newcastle-Ottawa Scale (NOS), which assigned a total of 9 stars to 8 items [25]. Studies awarded 6 or more stars were considered as high quality. The literature search, selection, data extraction and quality assessment were performed by two independent researchers (HJ and HL). Discrepancies were resolved by discussion.

### Definition of MMP2/MMP9 overexpression

MMP2/MMP9 overexpression in tumor sections was assessed using specific cut-offs of percentage of stained cells or the stained index (SI) that combines both percentage and intensity of staining, or the other methods. The SI was calculated as either the sum or product of staining percentage and intensity scores or determined using other complex scoring methods.

### Survival outcomes

The survival outcomes we investigated included overall survival (OS), disease-free survival (DFS), and recurrence-free survival (RFS). We obtained HR and 95% CI from univariate and/or multivariate analysis of associations between MMP2/MMP9 overexpression and survival. If no HR data were reported, we extracted survival data from the survival curves by using Engauge Digitizer software (https://github.com/markummitchell/engauge-digitizer) and estimated the HR and 95% CI by using the method by Tierney et al [26]. If a study reported HRs from both univariate and multivariate analysis, the latter was included in the overall analysis, and both were included in the subgroup analysis of univariate or multivariate analysis, respectively.

### Clinicopathological features

The clinicopathological features investigated in our analysis included tumor size, lymph node metastasis, distant metastasis, estrogen receptor (ER) status, progesterone receptor (PR) status, human epidermal growth factor receptor 2 (HER2) status, TNM stage, and histological grade, as those are associated with the prognosis of BC.

### Statistical analysis

Between-study heterogeneity was determined by using I^2^ and Q test. If I^2^ was 50% and *P* value for Q test was > 0.10, a fixed-effect model was used. Otherwise, a random-effect model was used. For survivals, pooled HR and 95% CI were calculated, and subgroup analyses regarding ethnicity (Caucasians, Asians), IHC analysis standard (percentage, SI, other cut-offs), HR data source (reported, estimated), analysis model (univariate, multivariate), sample size, cancer subtype, tissue (whole tissue, tissue microarray), IHC antibody (monoclonal, polyclonal) and antibody source (mouse, rabbit) were performed. For clinicopathological features, pooled OR and 95%CI were calculated, and subgroup analyses were also performed. Sensitivity analysis and meta-regression were performed to find the potential source of heterogeneity. Publication bias was assessed by funnel plot and Egger’s test. All the analyses were performed by using STATA 12.0 (Stata Corporation, TX, USA). *P* < 0.05 was considered statistically significant.

## Results

### Description of eligible studies

**Fig. 1:**
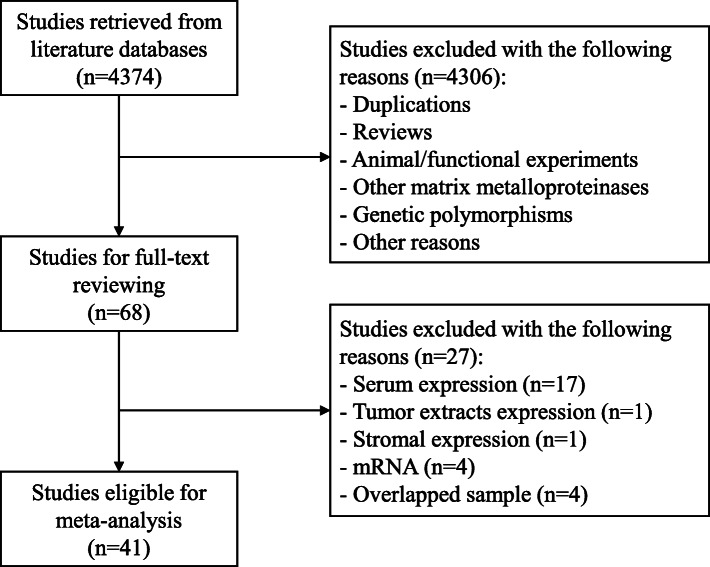
Flowchart of literature search and selection of eligible studies

The literature search yielded a total of 68 studies for full-text reviewing. Then, 27 studies were excluded as they detected MMP2/MMP9 expression in serum (*n* = 17) or cytosol tumor extracts (*n* = 1) or in stromal cells (*n* = 1), investigated mRNA expression (*n* = 4), duplicated with others (*n* = 4). Finally, a total of 41 studies comprising 6517 patients with primary breast cancers [16–23, 27–59] were included in our meta-analysis (Fig. 1). Among them, 31 studies with 4895 patients were eligible for survival analysis and 30 studies with 4743 cases for the analysis of clinicopathological features. The sample sizes range from 41 to 675. Regarding ethnicity, 22 studies were conducted in Caucasian populations and 19 in Asian populations. Regarding the definition of overexpression, 19 studies used percentage criteria, 19 used SI criteria and 3 used the other criteria [57–59]. According to NOS, 12 studies had 6 stars, 20 had 7 stars and 9 had 8 stars, indicating that all studies were of high quality.

For survival analysis, the HR and 95% CI were estimated from survival curves in 10 studies and were directly reported in 21 studies. Overexpression of MMP2 and MMP9 were investigated in 17 and 21 studies, respectively. The associations between MMP2 and MMP9 overexpression and clinicopathological features were reported in 14 and 20 studies, respectively. The characteristics of survival analysis and clinicopathological features were summarized in Table 1 and Table 2, respectively. The primary anti-huamn-MMP2/9 antibody used for IHC staining varied between studies and was summarized in Table S1.

**Table 1:** Characteristics of eligible studies for survival analysis

Author	Year	Country	Sample size	Percent of IDC (%)	Cut-off^a^	Follow-up	Protein	Survival outcome	HR data	Survival analysis	NOS
Talvensaari-Mattila [51]	1998	Finland	169	82	*P* > 0%	92 months	MMP2	OS	Rep	U	8
Talvensaari-Mattila [50]	1999	Finland	108	96	*P* > 0%	2 years	MMP2	RFS	Est	U	8
Talvensaari-Mattila [49]	2001	Finland	100	79	*P* > 0%	44 months	MMP2	OS, RFS	Est	U	7
Scorilas	2011	Greece	210	75	HSCORE> 175	62 months	MMP9	OS, RFS	Rep	U, M	7
Djonov [48]	2002	Switzerland	75	61	SI ≥ 1	NR	MMP2	OS, DFS	Rep	M	6
Hirvonen [47]	2003	Finland	137	NR	*P* > 0%	10 years	MMP2	RFS	Est	U	6
Fan [45]	2003	China	66	86	SI ≥ 1	30.5 months	MMP2, MMP9	OS	Rep	M	6
Talvensaari-Mattila [44]	2003	Finland	453	75	*P* > 0%	60–150 months	MMP2	OS	Rep	M	8
Li [19]	2004	China	270	90	*P* > 0%	61 months	MMP2, MMP9	OS, RFS	Rep	U, M	6
Pellikainen [58]	2004	Finland	415	64	*P* > 85%^b^	55 months	MMP9	RFS	Rep	M	7
Rahko [23]	2004	Finland	168	75	*P* > 1%	7–111 months	MMP9	OS, DFS	Rep	U	7
Ban [52]	2004	China	60	100	SI ≥ 3	> 5 years	MMP2	OS	Est	U	7
Zhou [42]	2005	China	112	84	*P* > 5%	48 months	MMP2	OS	Rep	M	7
Zhang [38]	2008	China	263	100	SI ≥ 6	92.1 months	MMP2, MMP9	OS	Rep	U	6
Zhao [53]	2008	China	71	93	*P* > 10%	54.94 months	MMP9	OS	Est	U	6
Sullu [22]	2011	Turkey	140	100	SI ≥ 5	63.2 months	MMP9	OS, DFS	Est	U	8
Ranogajec [37]	2012	Croatia	138	59	SI ≥ 2	5 years	MMP2	OS	Est	U	7
Fernandez-Guinea [21]	2013	Spain	97	100	SI	> 5 years	MMP2, MMP9	RFS	Rep	U, M	6
Zhao [18]	2013	China	127	NR	SI ≥ 6	NR	MMP9	OS	Rep	M	6
Liu [17]	2013	China	189	89	*P* > 10%	NR	MMP9	OS	Rep	U, M	8
Zeng [36]	2013	China	253	NR	*P* > 20%	15 years	MMP9	OS, DFS	Rep	U, M	8
Merdad [20]	2014	Saudi Arabia	45	84	SI ≥ 2	52.1 months	MMP9	OS	Rep	U	7
Puzovic [35]	2014	Croatia	121	100	SI	80.6 months	MMP2, MMP9	OS, DFS	Rep	U	7
Min [32]	2014	Korea	177	100	SI ≥ 1, SI ≥ 5	NR	MMP2, MMP9	OS	Rep	M	7
Yousef [30]	2014	Canada	300	NR	SI ≥ 5	NR	MMP9	RFS	Est	U	6
Bottino [59]	2014	Brazil	60	1	MOD> 191	NR	MMP9	OS	Rep	U	7
Huang [55]	2014	China	147	86	*P* > 10%	45.6 months	MMP9	OS, DFS	Rep	U, M	6
Ramos [29]	2016	Brazil	44	77	*P* > 10%	NR	MMP2	OS, DFS	Est	U	7
Li [16]	2017	China	80	NR	*P* > 25%	NR	MMP2, MMP9	OS	Est	U	7
Yang [28]	2018	Korea	173	100	SI ≥ 2	NR	MMP9	OS, DFS	Rep	U, M	8
Zhang [56]	2019	China	127	NR	SI ≥ 6	NR	MMP9	OS	Rep	M	7

^a^Cut-off for overexpression of matrix metalloproteinases in tumor cells by immunohistochemistry

^b^Median value

*IDC* infiltrating ductal carcinoma, *P* percentage of stained cells, *SI* staining index considering both percentage and intensity of staining, *OS* overall survival, *DFS* disease-free survival, *RFS* recurrence-free survival, *Rep* reported in the text, *Est* estimated from survival curve, *U* univariate analysis, *M* multivariate analysis, *NR* not reported, *MOD* mean optical density

**Table 2:** Characteristics of eligible studies for clinicopathological features

Author	Year	Country	Sample size	Cut-off^a^	Protein	T	N	M	ER	PR	HER2	S	G	NOS
Talvensaari-Mattila [51]	1998	Finland	169	*P* > 1%	MMP2	✓	✓	✓	✓	✓		✓	✓	8
Talvensaari-Mattila [50]	1999	Finland	108	*P* > 1%	MMP2	✓			✓	✓			✓	8
Talvensaari-Mattila [49]	2001	Finland	100	*P* > 1%	MMP2	✓							✓	7
Scorilas	2011	Greece	210	HSCORE> 175	MMP9	✓	✓		✓	✓		✓	✓	7
Hirvonen [47]	2003	Finland	137	*P* > 1%	MMP2	✓			✓	✓		✓		6
Nakopoulou	2003	Greece	135	*P* > 10%	MMP2	✓			✓	✓		✓		6
Fan [45]	2003	China	66	SI ≥ 1	MMP2, MMP9	✓	✓		✓	✓		✓		6
Talvensaari-Mattila [44]	2003	Finland	453	*P* > 1%	MMP2	✓	✓	✓	✓	✓			✓	8
Li [19]	2004	China	270	*P* > 1%	MMP2, MMP9	✓			✓	✓		✓		6
Rahko [23]	2004	Finland	168	*P* > 1%	MMP9	✓			✓	✓		✓	✓	7
Ban [52]	2004	China	60	SI ≥ 3	MMP2		✓					✓		7
Sivula	2005	Finland	194	*P* > 20%	MMP2	✓	✓		✓	✓				7
Zhou [42]	2005	China	112	*P* > 5%	MMP2		✓	✓	✓	✓			✓	7
Mylona	2007	Greece	175	*P* > 20%	MMP9	✓	✓		✓	✓		✓	✓	6
Hao	2007	China	76	SI ≥ 5	MMP9	✓	✓		✓	✓		✓	✓	7
Wu	2008	China	60	*P* > 50%	MMP9	✓	✓		✓	✓		✓	✓	7
Sullu [22]	2011	Turkey	140	SI ≥ 5	MMP9	✓	✓	✓	✓	✓	✓		✓	8
Zhao [18]	2013	China	127	SI ≥ 6	MMP9	✓	✓					✓	✓	6
Zeng [36]	2013	China	253	*P* > 20%	MMP9	✓	✓		✓	✓	✓	✓		8
Wu	2014	China	41	SI ≥ 1	MMP9		✓		✓	✓	✓	✓	✓	7
Tang	2014	China	156	SI ≥ 6	MMP9	✓	✓		✓	✓	✓	✓	✓	8
Min [32]	2014	Korea	177	SI ≥ 1, SI ≥ 5	MMP2, MMP9	✓	✓		✓	✓	✓	✓	✓	7
Youssef	2014	Egypt	67	*P* > 10%	MMP9	✓	✓		✓	✓	✓	✓	✓	7
Huang [55]	2014	China	147	*P* > 10%	MMP9	✓						✓		6
Ramos [29]	2016	Brazil	44	*P* > 10%	MMP2	✓	✓	✓	✓	✓	✓	✓		7
Li [16]	2017	China	80	*P* > 25%	MMP2, MMP9	✓	✓					✓		7
Yang [28]	2018	Korea	173	SI ≥ 2	MMP9	✓	✓		✓	✓	✓	✓	✓	8
Zhang [56]	2019	China	127	SI ≥ 6	MMP9	✓	✓					✓	✓	7
Zhou [54]	2009	China	43	SI ≥ 3	MMP9	✓	✓					✓	✓	7
Joseph [27]	2020	British	675	SI	MMP9	✓	✓		✓	✓		✓	✓	8

^a^Cut-off for overexpression of matrix metalloproteinases in tumor cells by immunohistochemistry

*P* percentage of stained cells, *SI* staining index considering both percentage and intensity of staining, *T* tumor size, *N* lymph node status, *M* distant metastasis, *ER* estrogen receptor, *PR* progesterone receptor, *HER2* human epidermal growth factor receptor 2, *S* TNM stage, *G* histological grade

### MMP2 overexpression and survival

**Fig. 2:**
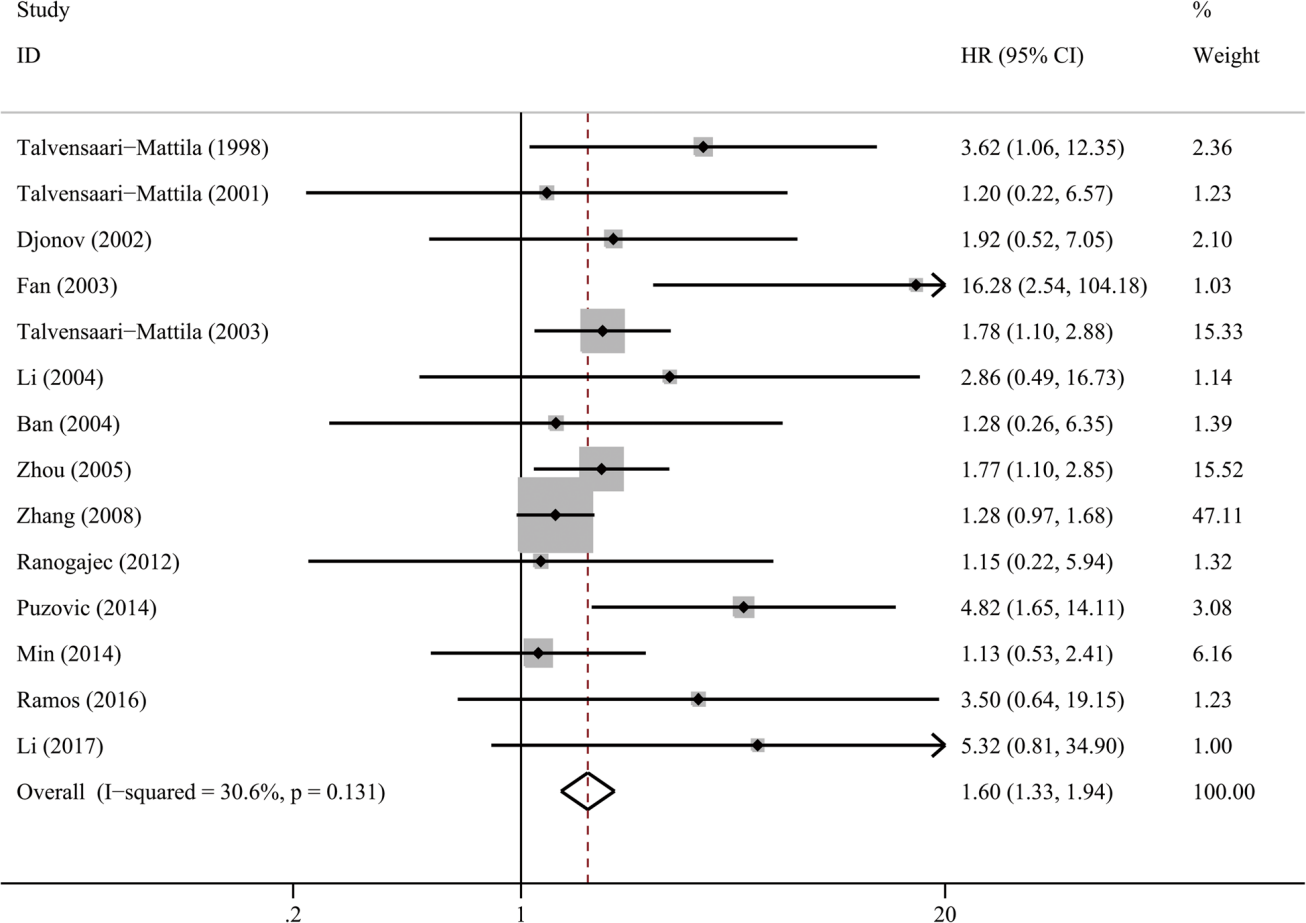
Forest plot of MMP2 overexpression with overall survival in patients with breast cancer

As shown in Table 3, there were no significant associations between MMP2 overexpression with DFS (HR = 1.79, *P* = 0.096) or RFS (HR = 1.21, *P* = 0.338) in BC. However, after pooling 14 studies, patients with MMP2 overexpression showed an unfavorable OS (HR = 1.60, 95% CI 1.33–1.94, *P* < 0.001, Fig. 2). The association was significant regarding ethnicity, IHC analysis standard, sample size and the anti-human-MMP2 antibody used for IHC staining (Table 3, Table S2). Subgroup of multivariate analysis adjusting HR for the other confounders (ER, PR, HER2, clinicopathological features) revealed that MMP2 overexpression was associated with unfavorable OS (HR = 1.78, 95%CI 1.32–2.39, *P* < 0.001) and may be an independent prognostic factor.

**Table 3:** Association between MMP2 overexpression and survival in patients with breast cancer

Survival	Subgroup	No. of studies	No. of patients	I^2^ (%)	P for heterogeneity	Pooled HR (95%CI)	P for effect size
DFS	Overall	3	240	0	0.724	1.79 (0.90–3.54)	0.096
OS	Overall	14	2128	30.6	0.131	1.60 (1.33–1.94)	< 0.001
	Ethnicity						
	Caucasians	7	1100	0	0.569	2.21 (1.47–3.06)	< 0.001
	Asians	7	1028	47.5	0.075	1.75 (1.14–2.70)	0.011
	IHC analysis standard						
	Percentage	7	1228	0	0.729	1.97 (1.46–2.68)	< 0.001
	SI	7	900	53.2	0.046	1.87 (1.08–3.26)	0.026
	HR data						
	Reported	9	1706	51.5	0.036	1.95 (1.37–2.79)	< 0.001
	Estimated	5	422	0	0.641	1.90 (0.89–4.07)	0.097
	Analysis model						
	Univariate	9	1245	32.2	0.160	1.52 (1.19–1.94)	0.001
	Multivariate	6	1153	29.9	0.211	1.78 (1.33–2.39)	< 0.001
	Sample size						
	> 150	5	796	23.3	0.266	1.43 (1.15–1.78)	0.002
	≤150	9	1332	20.2	0.263	2.19 (1.53–3.13)	< 0.001
	Cancer subtype						
	IDC	4	621	48.0	0.123	1.35 (1.06–1.73)	0.017
RFS	Overall	5	712	0	0.892	1.21 (0.82–1.81)	0.338

*OS* overall survival, *DFS* disease-free survival, *RFS* recurrence-free survival, *IHC* immunohistochemistry, *IDC* infiltrating ductal carcinoma, *SI* staining index, *HR* hazard ratio

### MMP9 overexpression and survival

**Fig. 3:**
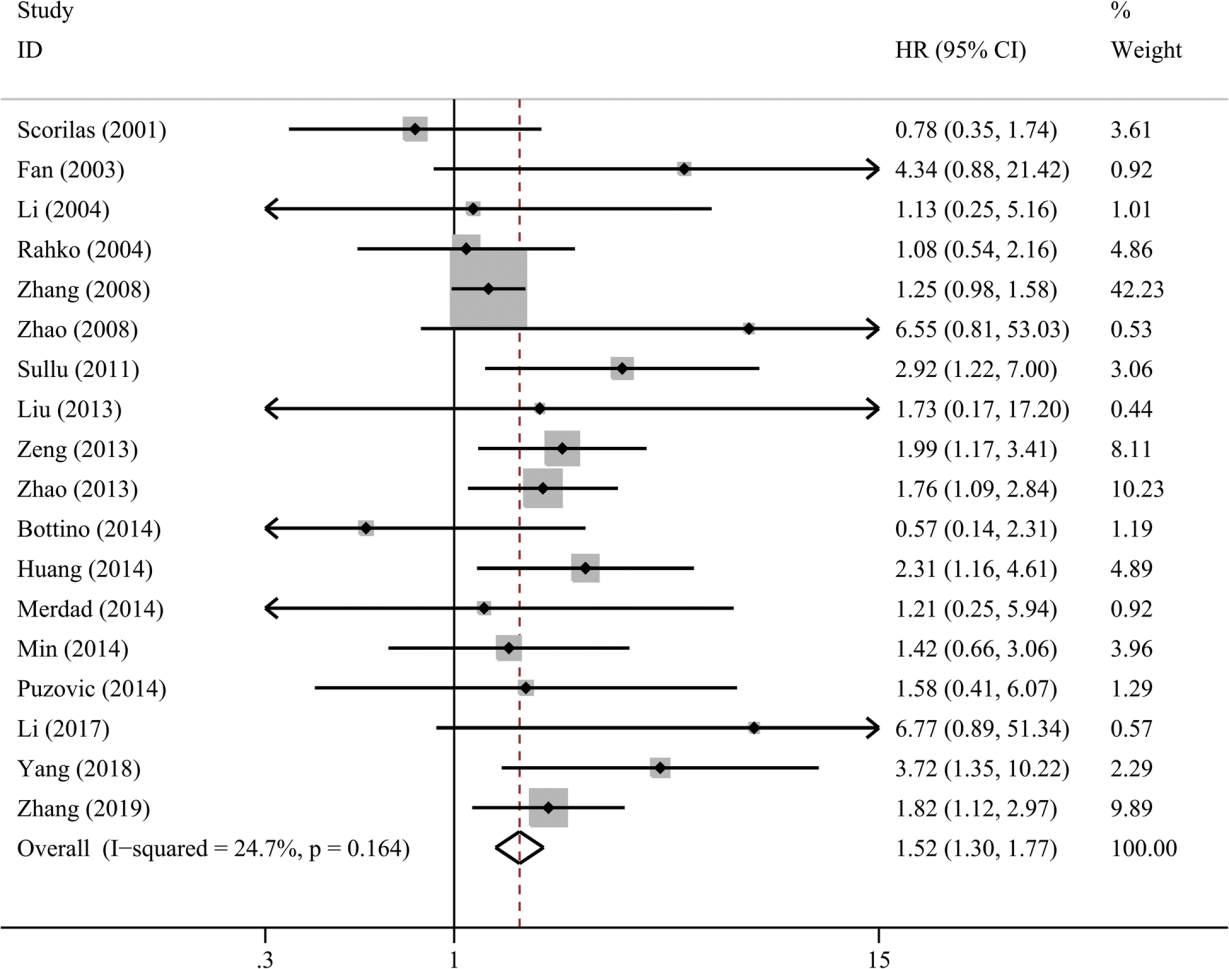
Forest plot of MMP9 overexpression with overall survival in patients with breast cancer

We found an association between MMP9 overexpression and DFS that almost reached significance (HR = 1.73, 95%CI 0.99–3.01, *P* = 0.052), whereas subgroup of univariate and multivariate analysis both suggested a shorter DFS (*P* = 0.034 and 0.006, respectively). MMP9 overexpression was not associated with RFS (HR = 1.53, 95%CI 0.73–3.18, *P* = 0.259, I^2^ = 79.7%) when we used a random-effect model. For OS, we pooled 18 studies with 2687 patients together by a fixed-effect model. The pooled HR was 1.52 (95% CI 1.30–1.77, *P* < 0.001, Fig. 3), suggesting an unfavorable OS in patients with overexpressed MMP9. A significantly shorter OS with MMP9 overexpression was found in Asian patients (HR = 1.58, 95% CI 1.34–1.86, *P* < 0.001) but not in Caucasian patients (*P* = 0.344) which may be due to small sample size (*n* = 744). The association was also significant in all of the other subgroup analyses regarding IHC analysis standard, analysis model, HR data source, sample size, tissue and antibody used for IHC analysis (Table 4, Table S2). In addition, MMP9 overexpression was correlated with unfavorable OS in patients with IDC (HR = 1.37, 95% CI 1.11–1.68, *P* = 0.003) and triple-negative breast cancer (TNBC) (HR = 1.88, 95% CI 1.39–2.55, *P* < 0.001).

**Table 4:** Association between MMP9 overexpression and survival in patients with breast cancer

Survival	Subgroup	No. of studies	No. of patients	I^2^ (%)	P for heterogeneity	Pooled HR (95%CI)	P for effect size
DFS	Overall	6	1002	70.5	0.005	1.73 (0.99–3.01)	0.052
	Analysis model						
	Univariate	6	1002	74.4	0.002	1.86 (1.05–3.31)	0.034
	Multivariate	3	573	56.7	0.100	2.73 (1.33–5.61)	0.006
OS	Overall	18	2687	24.7	0.164	1.52 (1.30–1.77)	< 0.001
	Ethnicity						
	Caucasians	6	744	22.0	0.268	1.21 (0.81–1.80)	0.344
	Asians	12	1943	25.0	0.198	1.58 (1.34–1.86)	< 0.001
	IHC analysis standard						
	Percentage	7	1178	2.8	0.404	1.85 (1.32–2.59)	< 0.001
	SI	9	1239	24.4	0.226	1.51 (1.26–1.80)	< 0.001
	Other cut-offs	2	270	0	0.703	0.72 (0.36–1.45)	0.359
	HR data						
	Reported	14	2249	9.9	0.344	1.43 (1.21–1.67)	< 0.001
	Estimated	4	438	0	0.644	2.84 (1.71–4.73)	< 0.001
	Analysis model						
	Univariate	14	2190	63.8	0.001	1.79 (1.24–2.59)	0.002
	Multivariate	9	1592	1.4	0.422	1.75 (1.37–2.22)	< 0.001
	Sample size						
	> 150	8	1703	18.8	0.281	1.33 (1.10–1.61)	0.003
	≤150	10	984	0	0.494	1.97 (1.51–2.56)	< 0.001
	Cancer subtype						
	IDC	6	934	43.3	0.116	1.37 (1.11–1.68)	0.003
	TNBC	4	590	0	0.933	1.88 (1.39–2.55)	< 0.001
RFS	Overall	5	1292	79.7	0.001	1.53 (0.73–3.18)	0.259

*OS* overall survival, *DFS* disease-free survival, *RFS* recurrence-free survival, *IHC* immunohistochemistry, *IDC* infiltrating ductal carcinoma, *TNBC* triple-negative breast cancer, *SI* staining index, *HR* hazard ratio

### MMPs overexpression and clinicopathological features

**Fig. 4:**
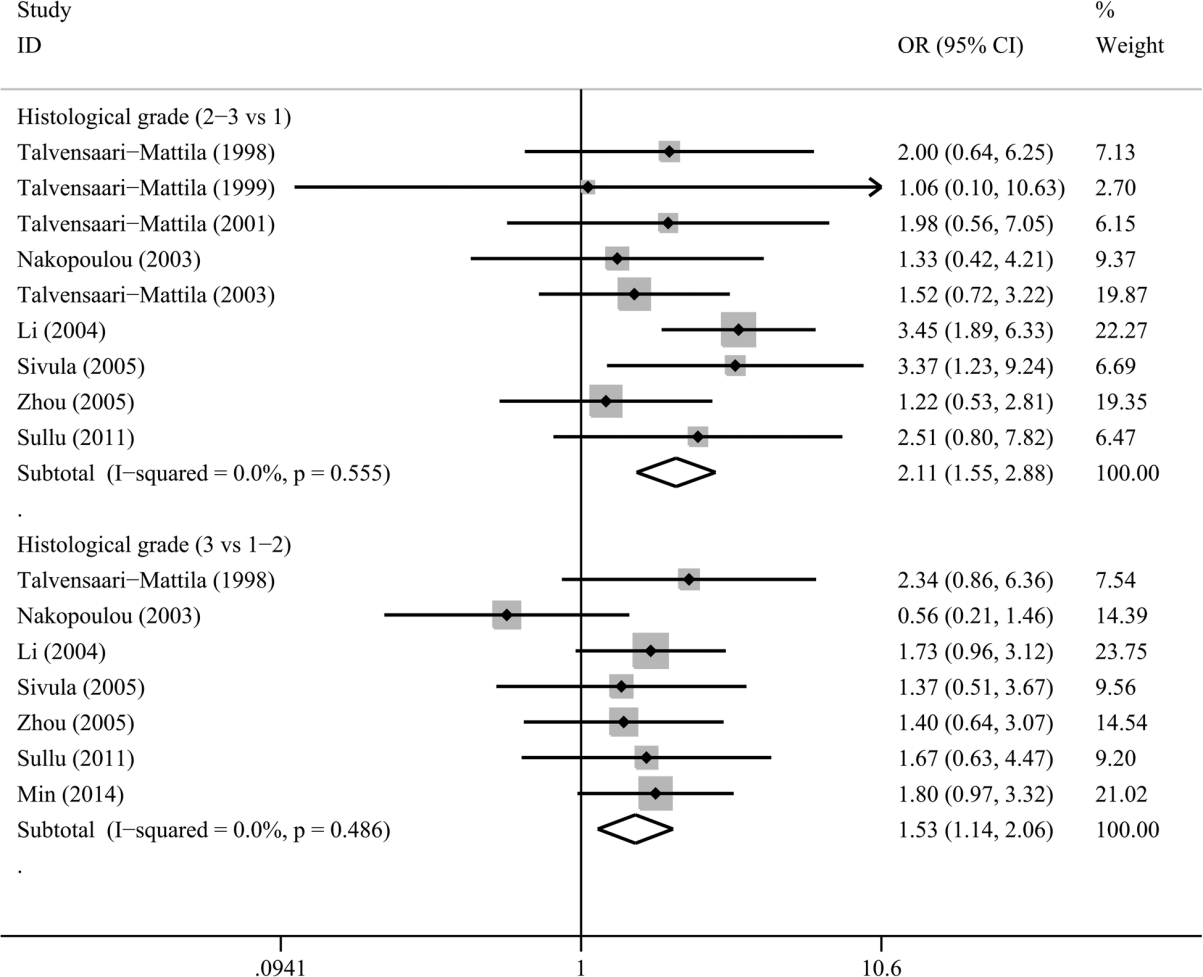
Forest plot of MMP2 overexpression with histological grade in patients with breast cancer

**Fig. 5:**
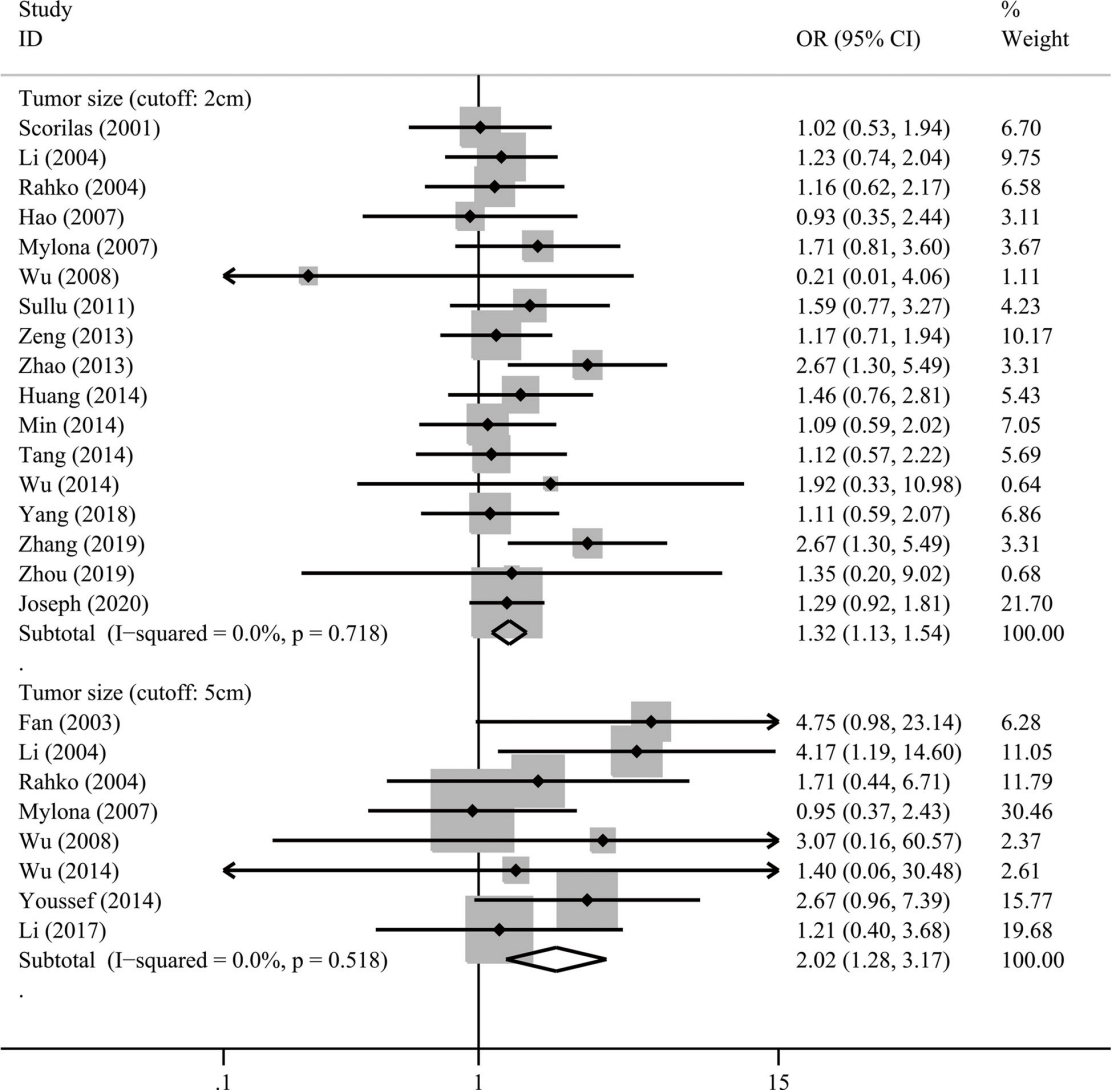
Forest plot of MMP9 overexpression with tumor size in patients with breast cancer

We investigated the associations between MMP2/MMP9 overexpression and clinicopathological features (Table 5, Table S3, Table S4). MMP2 overexpression was significantly associated with higher histological grades (for grade 2–3 vs 1, OR = 2.11, *P* < 0.001; for grade 3 vs. 1–2, OR = 1.53, *P* = 0.005; Fig. 4), higher tumor stages (OR = 2.09, *P* = 0.001) and distant metastasis (OR = 2.69, *P* = 0.005), but not with the other clinicopathological features. Meanwhile, MMP9 overexpression was found to be associated with higher histological grades (grade 3 vs. 1–2, OR = 1.77, *P* < 0.001), larger tumor size (for > 2 cm vs. ≤2 cm, OR = 1.32, 95% CI 1.13–1.54, *P* < 0.001; for > 5 cm vs. ≤5 cm, OR = 2.02, 95%CI 1.28–3.17, *P* = 0.002; Fig. 5), lymph node metastasis (OR = 2.90, *P* < 0.001), and positive HER2 (OR = 1.41, *P* = 0.021).

**Table 5:** Association between MMP2/9 overexpression and clinicopathological features in breast cancer patients

Clinicopathological feature	MMP2					MMP9				
	No. of patients	I^2^ (%)	Model	Pooled OR (95%CI)	*P*	No. of patients	I^2^ (%)	Model	Pooled OR (95%CI)	*P*
Tumor size (> 2 cm vs ≤2 cm)	1254	48.4	R	1.17 (0.78–1.75)	0.448	3005	0	F	1.32 (1.13–1.54)	< 0.001
Tumor size (> 5 cm vs ≤5 cm)	1286	15.9	F	1.12 (0.76–1.64)	0.568	924	0	F	2.02 (1.28–3.17)	0.002
Lymph node status (+ vs -)	1606	40.3	R	1.22 (0.88–1.70)	0.225	1945	77.1	R	2.90 (1.86–4.53)	< 0.001
Distant metastasis (+ vs -)	219	22.0	F	2.69 (1.35–5.39)	0.005	–	–	–	–	–
ER (+ vs -)	1784	47.1	R	0.82 (0.57–1.18)	0.290	1975	58.2	R	1.00 (0.71–1.39)	0.990
PR (+ vs -)	1660	5.7	F	1.07 (0.85–1.35)	0.545	1876	55.2	R	1.00 (0.73–1.38)	0.991
HER2 (+ vs -)	361	64.8	R	1.28 (0.49–3.37)	0.612	1007	0	F	1.41 (1.05–1.90)	0.021
TNM stage (III-IV vs I-II)	666	28.0	F	2.09 (1.36–3.21)	0.001	2419	70.7	R	2.00 (1.26–3.19)	0.004
Grade (2–3 vs 1)	1437	0	F	2.11 (1.55–2.88)	< 0.001	2051	61.9	R	1.55 (0.91–2.62)	0.107
Grade (3 vs 1–2)	1089	0	F	1.53 (1.14–2.06)	0.005	2609	48.4	R	1.77 (1.32–2.36)	< 0.001

*ER* estrogen receptor, *PR* progesterone receptor, *HER2* human epidermal growth factor receptor 2, *F* fixed effect model, *R* random effect model, *OR* odds ratio

+: positive; −: negative

### Sensitivity analysis and meta-regression

Sensitivity analysis revealed that excluding a single study did not obviously change the pooled effect size. Meta-regression showed that sample size had a significant impact on the association of PR status with MMP9 overexpression (*P* = 0.025) and ER status with MMP2 overexpression (*P* = 0.004).

### Publication bias

We observed significant publication bias in the analysis of the association of MMP2 overexpression with survival (*P* < 0.05), ER status (*P* = 0.003), and lymph node metastasis (*P* = 0.003), as well as MMP9 overexpression with lymph node status (*P* = 0.045) and TNM stage (*P* = 0.042). In the other analyses, the funnel plots were symmetric and *P* values of Egger’s test were > 0.05, indicating there was no obvious publication bias.

## Discussion

MMP2 and MMP9, also known as Gelatinase A and B, play key roles in the carcinogenesis of BC, with functions in cell proliferation, inflammation, angiogenesis, tumor invasion and metastasis [60]. However, studies on the potential associations between MMP2/9 expression and clinicopathological features and survival in BC have yielded conflicting results. Here, we performed a meta-analysis including 41 studies with 6517 BC patients and evaluated the prognostic values of MMP2/9 expression in tumor cells for BC. We found that MMP2 overexpression was significantly associated with shorter OS while MMP9 overexpression was related to shorter DFS and OS, indicating that MMP2 and MMP9 may serve as promising prognostic biomarkers for the treatment and management of BC patients.

Tumor metastasis is a crucial event of BC that severely affects the survival of patients, and may influence the determination of appropriate therapeutic strategies [61]. Overproduction of MMP2//9 induces the degradation of the major components of ECM and BM, allowing the escape of tumor cells and promoting subsequent metastasis [62]. Higher expression of MMP2/9 was found in tumor tissues compared with adjacent normal tissues [16]. In present study, MMP2 overexpression was associated with higher risk of distant metastasis and MMP9 overexpression correlated with lymph node metastasis, suggesting MMP2/9 may be indicators for BC metastasis. Moreover, both markers were associated with advanced clinical stages and poor tumor differentiation of BC. Therefore, MMP2/9 may be markers for poor prognosis and the detection of MMP2/9 protein expression may help determine strategies for treatment and follow-ups.

As described above, MMP2/9 overexpression has been associated with tumor size, metastasis, clinical stages and histological grades, all of which were well-known clinicopathological features influencing the survivals of BC patients. Therefore, the hazards ratio may be biased in univariate analysis and should be adjusted for these confounders using multivariate methods to investigate the independent roles of MMP2/9. In present analysis, subgroup of multivariate analysis demonstrated that both MMP2 and MMP9 overexpression predicted a significantly shorter OS after adjustment for known prognostic markers including ER, PR, HER2, and other clinicopathological features, indicating that MMP2 and MMP9 were independent predictors for survival of BC patients. Thus, MMP2/MMP9 overexpression with independent prognostic values may help make strategies for treatment and management of BC alone or together with known markers.

While the predictive roles of MMP2 and MMP9 were separately investigated in our meta-analysis, the role of MMP2 and MMP9 co-expression were not studied because of the small number of eligible studies. Li et al [19] reported that MMP2/9 co-expression was associated with shorter RFS in univariate and multivariate analyses but not with OS. Whereas, Puzovic et al [35] did not find associations of MMP2/9 co-expression with DFS and OS in BC patients. Since MMP2 and MMP9 belong to the same subtype of matrix metalloproteinases and share similar mechanism in promoting carcinogenesis, it is necessary to explore the prognostic value of the co-expression of both proteins in BC patients.

The present study mainly focused on the protein expression in tumor cells and has excluded MMP2/9 mRNA and protein expression in serum and stromal cells. Several studies detected serum MMP2/9 expressions by ELISA and correlated them with survival outcomes in BC patients [63–66]. However, the optimal cutoffs for high- and low-expression were mostly established using the median values, which varied among studies and were largely dependent on the enrolled samples. Thus, it was not suitable to pool these studies together or with the studies investigating protein expression derived from tumor cells. More efforts are needed to establish the optimal cutoff for serum expression of MMP2/9.

Some studies detected MMP2/9 expression in stromal cells by semi-quantitative analysis with IHC [21, 32, 41]. MMP2/9 are mainly expressed by neoplastic cells but also are derived from non-neoplastic stromal and inflammatory cells [67, 68]. Stromal MMP2/9 may also participate in tumor tissue remodeling and contribute to cancer progression [69, 70]. Min et al [32] found that stromal but not tumoral MMP2 was an independent predictive factor of OS, implying different prognostic roles of tumor- and stroma-derived MMP2 in BC. Mylona et al [41] reported significant associations of stromal MMP9 with poor OS and DFS. However, the prognostic value of stromal MMP2/9 in BC requires further investigation. Because the percentages of MMP2/9 overexpression were much higher in tumor cells compared with in stromal cells under the same IHC criteria [32, 41], we focused on tumoral MMP2/9 and excluded stromal MMP2/9 to keep the homogeneity of eligible studies in present study.

IDC is the most common subtype of BC [71]. Subgroup analysis demonstrated that MMP2/9 overexpression predicted significantly shorter OS in patients with IDC. TNBC is featured by the lack of ER, PR, and HER2 expression and comprises almost one-fifth of BC cases [72] and new prognostic indicators and treatment approaches for TNBC are urgently needed. Our analysis demonstrated that MMP9 overexpression was associated with poorer OS, larger tumor size, and higher TNM stage in TNBC, suggesting the promising role of MMP9 in the prognosis of TNBC.

There are some limitations in our study. Firstly, the HR and corresponding 95% CI in some studies were estimated from survival curves, which may deviate from the true values and affect the pooled effect sizes. For example, a significant association between MMP2 and OS was found in the subgroup with reported data but not in the subgroup with estimated data. To minimize the inaccuracy, two researchers independently extracted the data from survival curves. Secondly, there is currently no consensus on the threshold for MMPs overexpression by IHC. The cut-off values for percentage or staining index differ between studies, resulting in inconsistent positivity rates and predictive values of MMPs overexpression. This may be an important source of heterogeneity and limit the clinical use of MMP expression for the prediction of BC prognosis. Thirdly, we found obvious publication bias in the analysis of MMP overexpression associated with survival. The bias may potentially come from studies with univariate analysis (Egger’s test, *P* < 0.05) but not multivariate analysis (Egger’s test, *P* > 0.05), since studies with negative results of univariate analysis may tend to be unpublished.

## Conclusions

Our meta-analysis demonstrated that MMP2 and MMP9 overexpression in tumor cells was associated with poor survival, larger tumor size, lymph node metastasis, distant metastasis, higher clinical stage, and histological grade in patients with BC. These results suggest that MMP2 and MMP9 are potential markers for the prediction of BC prognosis.

## Supplementary Information

**Additional file 1: Table S1.** Tissues and antibodies for immunohistochemistry. **Table S2.** Subgroup analysis of overall survival stratified by immunohistochemistry antibody. **Table S3.** Subgroup analysis of association between MMP2 overexpression and clinicopathological features in breast cancer patients. **Table S4.** Subgroup analysis of association between MMP9 overexpression and clinicopathological features in breast cancer patients.

## Data Availability

The datasets used and/or analyzed during the current study are available from the corresponding author on reasonable request.

## Abbreviations

MMP: Matrix metalloproteinases
OS: Overall survival
DFS: Disease-free survival
RFS: Recurrence-free survival
HR: Hazard ratio
OR: Odds ratio
BC: Breast cancer
IDC: Infiltrating ductal cancer
TNBC: Triple-negative breast cancer
IHC: Immunohistochemistry
