# Supplementary material for: Prognostic values of tumoral MMP2 and MMP9 overexpression in breast cancer: a systematic review and meta-analysis
Source: BMC Cancer. 2021 Feb 10;21:149. doi: 10.1186/s12885-021-07860-2 (PMC7877076; doi:10.1186/s12885-021-07860-2)
Supplement: Supplementary file 1 — Additional file 1: Table S1. Tissues and antibodies for immunohistochemistry. Table S2. Subgroup analysis of overall survival stratified by immunohistochemistry antibody. Table S3. Subgroup analysis of association between MMP2 overexpression and clinicopathological features in breast cancer patients. Table S4. Subgroup analysis of association between MMP9 overexpression and clinicopathological features in breast cancer patients. [file 12885_2021_7860_MOESM1_ESM.docx]

Table S1 Tissues and antibodies for immunohistochemistry

| Study | Tissue | Anti-human-MMP2 antibody | | Anti-human-MMP9 antibody | |
| --- | --- | --- | --- | --- | --- |
|  |  | Antibody (dilution) | Manufacturer | Antibody (dilution) | Manufacturer |
| Talvensaari-Mattila (1998) | Whole tissue | Mouse monoclonal antibody | Diabor Ltd., Oulu, Finland | - | - |
| Talvensaari-Mattila (1999) | Whole tissue | Mouse monoclonal antibody | Diabor Ltd., Oulu, Finland | - | - |
| Talvensaari-Mattila (2001) | Whole tissue | Mouse monoclonal antibody | Diabor Ltd., Oulu, Finland | - | - |
| Djonov (2002) | Whole tissue | Mouse polyclonal antibody | NeoMarkers, CA, USA | - | - |
| Hirvonen (2003) | Whole tissue | Mouse monoclonal antibody | Diabor Ltd., Oulu, Finland | - | - |
| Nakopoulou (2003) | Whole tissue | Polyclonal antibody (1:100) | Not specified | - | - |
| Fan (2003) | Whole tissue | Mouse monoclonal antibody | Maixin Bio., Fujian, China | Mouse monoclonal antibody | Maixin Bio., Fujian, China |
| Talvensaari-Mattila (2003) | Whole tissue | Mouse monoclonal antibody | Diabor Ltd., Oulu, Finland | - | - |
| Li (2004) | Whole tissue | Rabbit polyclonal antibody (1:150) | Santa Cruz Bio., CA, USA | Rabbit polyclonal antibody (1:150) | Santa Cruz Bio., CA, USA |
| Rahko (2004) | Whole tissue | - | - | Mouse monoclonal antibody | Santa Cruz Bio., CA, USA |
| Ban (2004) | Whole tissue | Mouse monoclonal antibody | Maixin Bio., Fujian, China | - | - |
| Sivula (2005) | Tissue microarray | Mouse monoclonal antibody | Diabor Ltd., Oulu, Finland | - | - |
| Zhou (2005) | Whole tissue | Monoclonal antibody (1:200) | Santa Cruz Bio., CA, USA | - | - |
| Mylona (2007) | Whole tissue | - | - | Rabbit polyclonal antibody (1:60) | Chemicon Intertional Inc., Temecula, CA, USA |
| Hao (2007) | Whole tissue | - | - | Mouse monoclonal antibody | NeoMarkers, CA, USA |
| Wu (2008) | Whole tissue | - | - | Monoclonal antibody (1:100) | Santa Cruz Bio., Santa Cruz, CA, USA |
| Zhang (2008) | Tissue microarray | Polyclonal antibody (1:150) | NeoMarkers, CA, USA | Polyclonal antibody (1:150) | NeoMarkers, CA, USA |
| Zhao (2008) | Whole tissue | - | - | Mouse monoclonal antibody | Santa Cruz Bio., CA, USA |
| Zhou (2019) | Whole tissue | - | - | Mouse monoclonal antibody | Not specified |
| Sullu (2011) | Whole tissue | Rabbit polyclonal antibody (1:50) | Spring Bioscience, CA, USA | Rabbit polyclonal antibody (1:100) | Neomarkers, CA, USA |
| Ranogajec (2012) | Whole tissue | Mouse monoclonal antibody | Novocastra, UK | - | - |
| Fernandez-Guinea (2013) | Tissue microarray | Polyclonal antibody (1:50) | Abcam Cambridge, UK | Not specified | NeoMarkers, CA, USA |
| Zhao (2013) | Whole tissue | - | - | Rabbit polyclonal antibody (1:100) | Zhongshan Bio., Beijing, China |
| Liu (2013) | Whole tissue | - | - | Not specified | Not specified |
| Zeng (2013) | Whole tissue | - | - | Mouse monoclonal antibody | Calbiochem |
| Merdad (2014) | Whole tissue | - | - | Rabbit polyclonal antibody | Spring Bioscience, Fremont, CA, USA |
| Puzovic (2014) | Tissue microarray | Not specified | Novocastra, UK | Not specified | Novocastra, UK |
| Wu (2014) | Whole tissue | - | - | Not specified (1:50) | Abcam Cambridge, UK |
| Tang (2014) | Whole tissue | - | - | Rabbit polyclonal antibody (1:100) | Boster Bio, Wuhan, China |
| Min (2014) | Tissue microarray | Rabbit polyclonal antibody (1:100) | NeoMarkers, CA, USA | Rabbit polyclonal antibody (1:100) | NeoMarkers, CA, USA |
| Youssef (2014) | Whole tissue | - | - | Mouse monoclonal antibody (1:200) | Thermo Fisher Scientific Inc., Fremont, CA, USA |
| Yousef (2014) | Tissue microarray | - | - | Polyclonal antibody (1:100) | Abcam, Canada |
| Huang (2014) | Whole tissue | - | - | Not specified | Santa Cruz Bio., CA, USA |
| Ramos (2016) | Whole tissue | Rabbit polyclonal antibody (1:400) | MyBioSource | - | - |
| Li (2017) | Whole tissue | Rabbit polyclonal antibody | Proteintech Group, Inc., Wuhan, China | Rabbit polyclonal antibody | Proteintech Group, Inc., Wuhan, China |
| Yang (2018) | Tissue microarray | - | - | Rabbit polyclonal antibody (1:100) | NeoMarkers, CA, USA |
| Zhang (2019) | Whole tissue | - | - | Rabbit polyclonal antibody (1:100) | Zhongshanjinqiao Bio., Beijing, China |
| Joseph (2020) | Tissue microarray | - | - | Rabbit monoclonal antibody (1:100) | Abcam |

Table S2 Subgroup analysis of overall survival stratified by immunohistochemistry antibody

| Subgroup | No. of studies | No. of patients | I^2^ (%) | P for heterogeneity | Pooled HR (95%CI) | P for effect size |
| --- | --- | --- | --- | --- | --- | --- |
| MMP2 |  |  |  |  |  |  |
| Primary antibody |  |  |  |  |  |  |
| Monoclonal | 7 | 1098 | 17.1 | 0.229 | 1.89 (1.39-2.57) | <0.001 |
| Polyclonal | 6 | 909 | 0 | 0.468 | 1.36 (1.06-1.74) | 0.014 |
| Antibody source |  |  |  |  |  |  |
| Mouse | 7 | 1061 | 15.6 | 0.311 | 1.98 (1.35-2.89) | <0.001 |
| Rabbit | 4 | 571 | 15.5 | 0.314 | 1.72 (0.93-3.16) | 0.082 |
| Not specified | 3 | 496 | 68.2 | 0.043 | 1.80 (1.06-3.07) | 0.031 |
| Tissue |  |  |  |  |  |  |
| Whole tissue | 11 | 1567 | 0 | 0.534 | 1.99 (1.50-2.65) | <0.001 |
| Tissue microarray | 3 | 561 | 65.3 | 0.056 | 1.64 (0.85-3.16) | 0.139 |
| MMP9 |  |  |  |  |  |  |
| Primary antibody |  |  |  |  |  |  |
| Monoclonal | 4 | 558 | 38.2 | 0.139 | 1.78 (1.19-2.66) | 0.005 |
| Polyclonal | 10 | 1612 | 33.7 | 0.139 | 1.45 (1.22-1.73) | <0.001 |
| Not specified | 4 | 517 | 3.5 | 0.375 | 1.72 (1.00-2.98) | 0.051 |
| Antibody source |  |  |  |  |  |  |
| Mouse | 4 | 558 | 38.2 | 0.183 | 1.78 (1.19-2.66) | 0.005 |
| Rabbit | 8 | 1139 | 0 | 0.590 | 1.92 (1.46-2.51) | <0.001 |
| Not specified | 6 | 990 | 12.9 | 0.332 | 1.27 (1.03-1.56) | 0.027 |
| Tissue |  |  |  |  |  |  |
| Whole tissue | 14 | 1953 | 15.5 | 0.284 | 1.73 (1.39-2.14) | <0.001 |
| Tissue microarray | 4 | 734 | 31.4 | 0.224 | 1.33 (1.07-1.65) | 0.010 |

Table S3 Subgroup analysis of association between MMP2 overexpression and clinicopathological features in breast cancer patients

| Clinicopathological feature | No. of studies | No. of patients | I^2^ (%) | Model | Pooled OR | 95%CI | P |
| --- | --- | --- | --- | --- | --- | --- | --- |
| Distant metastasis (+ vs -) | 4 | 219 | 22.0 | F | **2.69** | **1.35-5.39** | **0.005** |
| ER (+ vs -) | 12 | 1784 | 47.1 | R | 0.82 | 0.57-1.18 | 0.290 |
| Caucasians | 8 | 1159 | 43.4 | R | 0.78 | 0.48-1.28 | 0.327 |
| Asians | 4 | 625 | 62.3 | R | 0.85 | 0.47-1.54 | 0.602 |
| Percentage | 9 | 1401 | 37.9 | F | 1.02 | 0.79-1.34 | 0.850 |
| SI | 3 | 383 | 51.9 | R | 0.55 | 0.26-1.15 | 0.111 |
| Sample>150 | 4 | 968 | 0 | F | 1.31 | 0.96-1.79 | 0.087 |
| Sample≤150 | 8 | 816 | 17.6 | F | **0.56** | **0.39-0.80** | **0.001** |
| Monoclonal antibody | 7 | 1018 | 30.4 | F | 0.86 | 0.60-1.21 | 0.380 |
| Polyclonal antibody | 5 | 766 | 66.2 | R | 0.82 | 0.44-1.52 | 0.531 |
| Grade (2-3 vs 1) | 9 | 1437 | 0 | F | **2.11** | **1.55-2.88** | **<0.001** |
| Caucasians | 7 | 1055 | 0 | F | **1.90** | **1.26-2.86** | **0.002** |
| Asians | 2 | 382 | 74.5 | R | **2.41** | **1.49-3.91** | **<0.001** |
| Percentage | 8 | 1297 | 0 | F | **2.11** | **1.55-2.88** | **<0.001** |
| Monoclonal antibody | 6 | 922 | 0 | F | **1.71** | **1.13-2.59** | **0.012** |
| Polyclonal antibody | 3 | 515 | 4.7 | F | **2.77** | **1.72-4.46** | **<0.001** |
| Grade (3 vs 1-2) | 7 | 1089 | 0 | F | **1.53** | **1.14-2.06** | **0.005** |
| Caucasians | 4 | 530 | 34.3 | F | 1.33 | 0.82-2.16 | 0.245 |
| Asians | 3 | 559 | 0 | F | **1.67** | **1.15-2.43** | **0.007** |
| Percentage | 5 | 772 | 20.1 | F | **1.44** | **1.00-2.06** | **0.049** |
| SI | 2 | 317 | 0 | F | **1.76** | **1.04-2.97** | **0.034** |
| Monoclonal antibody | 3 | 397 | 0 | F | 1.61 | 0.96-2.72 | 0.071 |
| Polyclonal antibody | 4 | 692 | 35.5 | F | **1.50** | **1.04-2.14** | **0.028** |
| HER2 (+ vs -) | 3 | 361 | 64.8 | R | 1.28 | 0.49-3.37 | 0.612 |
| IDC (vs other subtypes) | 6 | 1206 | 0 | F | 1.26 | 0.91-1.77 | 0.167 |
| Caucasians | 5 | 936 | 0 | F | 1.25 | 0.86-1.80 | 0.241 |
| Lymph node status (+ vs -) | 11 | 1606 | 40.3 | R | 1.22 | 0.88-1.70 | 0.225 |
| Caucasians | 6 | 1111 | 0 | F | 0.95 | 0.70-1.28 | 0.721 |
| Asians | 5 | 495 | 67.4 | F | 1.91 | 0.91-3.99 | 0.086 |
| Sample>150 | 4 | 992 | 0 | F | 0.86 | 0.63-1.17 | 0.333 |
| Sample≤150 | 7 | 614 | 26.1 | F | **1.59** | **1.10-2.29** | **0.013** |
| Percentage | 7 | 1186 | 18.9 | F | 1.10 | 0.83-1.46 | 0.517 |
| SI | 4 | 420 | 67.9 | R | 1.44 | 0.65-3.17 | 0.367 |
| Monoclonal antibody | 6 | 1053 | 48.8 | R | 1.42 | 0.90-2.25 | 0.134 |
| Polyclonal antibody | 5 | 553 | 31.8 | F | 0.96 | 0.66-1.39 | 0.815 |
| PR (+ vs -) | 11 | 1660 | 5.7 | F | 1.07 | 0.85-1.35 | 0.545 |
| Caucasians | 7 | 1035 | 32.3 | F | 0.98 | 0.71-1.36 | 0.913 |
| Asians | 4 | 625 | 0 | F | 1.17 | 0.85-1.61 | 0.337 |
| Percentage | 9 | 1417 | 11.1 | F | 1.00 | 0.77-1.28 | 0.971 |
| SI | 2 | 243 | 0 | F | 1.47 | 0.88-2.48 | 0.145 |
| Sample>150 | 5 | 1126 | 0 | F | 1.19 | 0.90-1.57 | 0.210 |
| Sample≤150 | 6 | 534 | 33.9 | F | 0.86 | 0.58-1.28 | 0.455 |
| Monoclonal antibody | 7 | 1034 | 0 | F | 1.09 | 0.80-1.50 | 0.577 |
| Polyclonal antibody | 4 | 626 | 54.6 | R | 1.02 | 0.58-1.80 | 0.937 |
| Tumor size (>2cm vs ≤2cm) | 8 | 1254 | 48.4 | R | 1.17 | 0.78-1.75 | 0.448 |
| Caucasians | 6 | 807 | 56.9 | R | 1.12 | 0.61-2.03 | 0.716 |
| Asians | 2 | 447 | 40.6 | F | 1.31 | 0.89-1.93 | 0.177 |
| Percentage | 6 | 943 | 61.1 | R | 1.19 | 0.67-2.12 | 0.561 |
| SI | 2 | 311 | 0 | F | 1.07 | 0.66-1.74 | 0.789 |
| Monoclonal antibody | 3 | 494 | 79.7 | R | 1.17 | 0.36-3.81 | 0.798 |
| Polyclonal antibody | 5 | 760 | 0 | F | 1.21 | 0.88-1.65 | 0.249 |
| Tumor size (>5cm vs ≤5cm) | 8 | 1286 | 15.9 | F | 1.12 | 0.76-1.64 | 0.568 |
| Caucasians | 5 | 870 | 0 | F | 0.91 | 0.57-1.45 | 0.695 |
| Asians | 3 | 416 | 62.6 | R | 1.45 | 0.46-4.59 | 0.526 |
| Percentage | 7 | 1220 | 27.9 | F | 1.14 | 0.76-1.70 | 0.534 |
| Monoclonal antibody | 5 | 892 | 0 | F | 0.85 | 0.54-1.34 | 0.491 |
| Polyclonal antibody | 3 | 394 | 58.9 | R | 1.89 | 0.55-6.46 | 0.311 |
| TNM stage (III-IV vs I-II) | 7 | 666 | 28.0 | F | **2.09** | **1.36-3.21** | **0.001** |
| Caucasians | 3 | 348 | 50.0 | F | 1.37 | 0.69-2.72 | 0.371 |
| Asians | 4 | 318 | 0 | F | **2.76** | **1.60-4.76** | **<0.001** |
| Percentage | 5 | 540 | 51.9 | R | **2.14** | **1.03-4.48** | **0.043** |
| SI | 2 | 126 | 0 | F | 2.05 | 0.77-5.43 | 0.149 |
| Monoclonal antibody | 4 | 407 | 0 | F | **2.34** | **1.35-4.04** | **0.002** |
| Polyclonal antibody | 3 | 259 | 73.5 | R | 2.09 | 0.49-9.01 | 0.321 |

ER: estrogen receptor; PR: progesterone receptor; HER2: human epidermal growth factor receptor 2; F: fixed effect model; R: random effect model; OR: odds ratio; SI: staining index; IDC: infiltrating ductal carcinoma; +: positive; -: negative.

Table S4 Subgroup analysis of association between MMP9 overexpression and clinicopathological features in breast cancer patients

| Clinicopathological feature | No. of studies | No. of patients | I^2^ (%) | Model | Pooled OR | 95%CI | P |
| --- | --- | --- | --- | --- | --- | --- | --- |
| ER (+ vs -) | 14 | 1975 | 58.2 | R | 1.00 | 0.71-1.39 | 0.990 |
| Caucasians | 5 | 703 | 64.5 | R | 0.87 | 0.45-1.69 | 0.682 |
| Asians | 9 | 1272 | 57.4 | R | 1.07 | 0.72-1.60 | 0.730 |
| Percentage | 6 | 978 | 68.8 | R | 0.89 | 0.51-1.54 | 0.674 |
| SI | 7 | 787 | 55.5 | R | 1.15 | 0.69-1.93 | 0.583 |
| Sample>150 | 8 | 1567 | 54.1 | R | 1.06 | 0.76-1.48 | 0.716 |
| Sample≤150 | 6 | 408 | 67.1 | R | 0.84 | 0.34-2.06 | 0.706 |
| Monoclonal antibody | 6 | 677 | 66.3 | R | 0.78 | 0.41-1.51 | 0.465 |
| Polyclonal antibody | 7 | 1257 | 62.5 | R | 1.10 | 0.72-1.68 | 0.667 |
| Grade (2-3 vs 1) | 12 | 2051 | 61.9 | R | 1.55 | 0.91-2.62 | 0.107 |
| Caucasians | 6 | 1388 | 72.2 | R | 1.24 | 0.55-2.83 | 0.606 |
| Asians | 6 | 663 | 46.2 | R | 2.03 | 1.00-4.13 | 0.051 |
| Percentage | 5 | 700 | 74.8 | R | 0.89 | 0.32-2.49 | 0.818 |
| SI | 6 | 1141 | 46.9 | R | **2.49** | **1.31-4.73** | **0.005** |
| Sample>150 | 5 | 1490 | 30.7 | F | **1.97** | **1.41-2.74** | **<0.001** |
| Sample≤150 | 7 | 561 | 73.4 | R | 1.25 | 0.38-4.12 | 0.712 |
| Monoclonal antibody | 6 | 1048 | 74.3 | R | 1.21 | 0.42-3.45 | 0.721 |
| Polyclonal antibody | 5 | 962 | 52.3 | R | **1.90** | **1.04-3.46** | **0.036** |
| Grade (3 vs 1-2) | 16 | 2609 | 48.4 | R | **1.77** | **1.32-2.36** | **<0.001** |
| Caucasians | 6 | 1388 | 74.0 | R | 1.78 | 0.96-3.32 | 0.068 |
| Asians | 10 | 1221 | 8.4 | F | **1.87** | **1.44-2.42** | **<0.001** |
| Percentage | 5 | 700 | 49.5 | R | 1.16 | 0.66-2.03 | 0.617 |
| SI | 10 | 1699 | 24.1 | F | **2.22** | **1.78-2.77** | **<0.001** |
| Sample>150 | 7 | 1823 | 0 | F | **1.94** | **1.56-2.41** | **<0.001** |
| Sample≤150 | 9 | 786 | 67.5 | R | 1.56 | 0.79-3.09 | 0.201 |
| Monoclonal antibody | 6 | 1048 | 65.8 | R | 1.27 | 0.63-2.58 | 0.503 |
| Polyclonal antibody | 9 | 1520 | 10.8 | F | **2.10** | **1.65-2.68** | **<0.001** |
| HER2 (+ vs -) | 7 | 1007 | 0 | F | **1.41** | **1.05-1.90** | **0.021** |
| Caucasians | 2 | 207 | 0 | F | **2.68** | **1.20-5.99** | **0.016** |
| Asians | 5 | 800 | 0 | F | 1.26 | 0.92-1.73 | 0.154 |
| Percentage | 2 | 320 | 67.3 | R | 1.56 | 0.43-5.61 | 0.496 |
| SI | 5 | 687 | 0 | F | **1.59** | **1.10-2.30** | **0.013** |
| Monoclonal antibody | 2 | 320 | 67.3 | R | **1.59** | **1.09-2.30** | **0.015** |
| Polyclonal antibody | 4 | 646 | 0 | F | 1.56 | 0.43-5.61 | 0.496 |
| Lymph node status (+ vs -) | 16 | 1945 | 77.1 | R | **2.90** | **1.86-4.53** | **<0.001** |
| Caucasians | 4 | 566 | 51.4 | R | 1.64 | 0.96-2.82 | 0.072 |
| Asians | 12 | 1379 | 78.6 | R | **3.55** | **2.02-6.24** | **<0.001** |
| Percentage | 5 | 632 | 54.7 | R | **2.58** | **1.45-4.61** | **0.001** |
| SI | 10 | 1103 | 81.6 | R | **3.37** | **1.72-6.61** | **<0.001** |
| Sample>150 | 6 | 1141 | 0 | F | **1.55** | **1.22-1.98** | **<0.001** |
| Sample≤150 | 10 | 804 | 72.4 | R | **4.88** | **2.44-9.74** | **<0.001** |
| Monoclonal antibody | 6 | 565 | 60.2 | R | **4.32** | **2.13-8.77** | **<0.001** |
| Polyclonal antibody | 9 | 1339 | 83.0 | R | **2.63** | **1.46-4.74** | **0.001** |
| IDC | 4 | 534 | 41.2 | F | **1.68** | **1.18-2.39** | **0.004** |
| IDC (vs other subtypes) | 4 | 664 | 0 | F | 0.95 | 0.61-1.47 | 0.820 |
| PR (+ vs -) | 13 | 1876 | 55.2 | R | 1.00 | 0.73-1.38 | 0.991 |
| Caucasians | 4 | 604 | 80.5 | R | 0.93 | 0.37-2.32 | 0.877 |
| Asians | 9 | 1272 | 29.4 | F | 1.07 | 0.85-1.35 | 0.546 |
| Percentage | 6 | 977 | 64.4 | R | 0.94 | 0.58-1.53 | 0.814 |
| SI | 6 | 689 | 0 | F | 0.83 | 0.60-1.14 | 0.440 |
| Sample>150 | 8 | 1566 | 50.7 | R | 1.17 | 0.86-1.60 | 0.324 |
| Sample≤150 | 5 | 310 | 42.2 | F | **0.56** | **0.33-0.95** | **0.032** |
| Monoclonal antibody | 6 | 676 | 62.7 | R | 0.75 | 0.41-1.35 | 0.332 |
| Polyclonal antibody | 6 | 1159 | 58.6 | R | 1.15 | 0.77-1.72 | 0.497 |
| Tumor size (>2cm vs ≤2cm) | 17 | 3005 | 0 | F | **1.32** | **1.13-1.54** | **<0.001** |
| Caucasians | 5 | 1335 | 0 | F | **1.29** | **1.02-1.64** | **0.036** |
| Asians | 12 | 1650 | 0 | F | **1.34** | **1.09-1.65** | **0.005** |
| Percentage | 6 | 1070 | 0 | F | 1.25 | 0.96-1.62 | 0.091 |
| SI | 10 | 1725 | 0 | F | **1.40** | **1.14-1.72** | **0.001** |
| Sample>150 | 9 | 2250 | 0 | F | **1.21** | **1.01-1.45** | **0.040** |
| Sample≤150 | 8 | 755 | 0 | F | **1.72** | **1.26-2.35** | **0.001** |
| Monoclonal antibody | 6 | 1270 | 0 | F | 1.19 | 0.93-1.52 | 0.161 |
| Polyclonal antibody | 9 | 1547 | 14.9 | F | **1.41** | **1.13-1.75** | **0.002** |
| TNBC | 4 | 444 | 0 | F | **2.09** | **1.41-3.08** | **<0.001** |
| Tumor size (>5cm vs ≤5cm) | 8 | 924 | 0 | F | **2.02** | **1.28-3.17** | **0.002** |
| Caucasians | 3 | 407 | 7.5 | F | 1.57 | 0.85-2.91 | 0.153 |
| Asians | 5 | 517 | 0 | F | **2.63** | **1.34-5.18** | **0.005** |
| Percentage | 6 | 547 | 0 | F | **1.85** | **1.14-2.99** | **0.013** |
| SI | 2 | 107 | 0 | F | 3.77 | 0.92-15.38 | 0.065 |
| Monoclonal antibody | 4 | 360 | 0 | F | **2.74** | **1.36-5.54** | **0.005** |
| Polyclonal antibody | 3 | 523 | 46..0 | F | 1.61 | 0.88-2.95 | 0.122 |
| TNM stage (III-IV vs I-II) | 15 | 2419 | 70.7 | R | **2.00** | **1.26-3.19** | **0.004** |
| Caucasians | 4 | 1074 | 40.3 | F | 1.45 | 0.94-2.23 | 0.090 |
| Asians | 10 | 1135 | 54.2 | R | **2.66** | **1.63-4.35** | **<0.001** |
| Percentage | 7 | 946 | 62.6 | R | **2.18** | **1.12-4.24** | **0.022** |
| SI | 7 | 1263 | 36.0 | F | **2.24** | **1.62-3.09** | **<0.001** |
| Sample>150 | 6 | 1626 | 70.4 | R | 1.04 | 0.59-1.83 | 0.888 |
| Sample≤150 | 9 | 793 | 0 | F | **3.56** | **2.45-5.19** | **<0.001** |
| Monoclonal antibody | 8 | 1400 | 53.8 | R | **1.97** | **1.06-3.66** | **0.031** |
| Polyclonal antibody | 6 | 872 | 83.6 | R | 1.83 | 0.80-4.16 | 0.150 |
| TNBC | 4 | 444 | 0 | F | **3.07** | **1.92-4.91** | **<0.001** |

ER: estrogen receptor; PR: progesterone receptor; HER2: human epidermal growth factor receptor 2; F: fixed effect model; R: random effect model; OR: odds ratio; SI: staining index; IDC: infiltrating ductal carcinoma. TNBC: triple-negative breast cancer; +: positive; -: negative;
